# Enlargements of the Liver.—IX

**Published:** 1910-03-05

**Authors:** 


### Medicine.

### ENLARGEMENTS OF THE LIVER.—IX.

### ABSCESSES OF THE LIVER.

The liver may be considerably enlarged by the presence within it of a single abscess. In whatever part the abscess forms it usually increases in size towards the surface and causes a local prominence or distinct bulging. In size the abscess may vary from that of a Tangerine orange or less to that of a child's head or more; it may contain several pints of pus. The latter is often of a typical anchovy sauce consistence and colour. Perihepatitis usually occurs over the surface of the abscess and in consequence the liver may become fixed to adjacent parts; the pleura upon the other side of the diaphragm from the abscess becomes similarly adherent; this has an important bearing both on the surgical treatment of the condition and also on the termination of the case, for it determines the point at which the abscess, if left to itself, may eventually open.

Single hepatic abscess is a condition most commonly found in people who have resided in the tropics, and it has often been preceded by dysentery due to the *amœba coli*. It may also follow violent blows in the hepatic region, and sometimes has been caused by foreign bodies such as needles, fish bones and the like that have invaded the liver from the œsophagus or stomach. Suppuration in a gumma, a hydatid cyst, or in a mass of growth in the liver, may cause a single abscess. An analysis of the cases of single abscess in a large general hospital shows that suppurating hydatid cyst is there the most frequent cause in this country of a large single collection of pus in the liver. In the Guy's Hospital Museum there is a specimen of a liver containing a very big abscess that arose as a direct sequela of scarlet fever.

The physical signs will depend entirely on the size and the position of the abscess. The right lobe is much more frequently affected than is the left, and of the right lobe the upper part chiefly, so that the abscess projects upwards, pushing the diaphragm before it, compressing the base of the right lung and simulating empyema. Particular care should always be taken in accurately mapping out the upper line of dulness, which will be found to have a distinctly convex outline in many cases of hepatic abscess with the convexity upwards and highest in the axilla, whereas in empyema the upper limit of the dulness will usually be straighter, in a line which starts highest posteriorly and thence runs obliquely downwards and forwards.

Owing to the upper surface of the liver becoming adherent to the under surface of the diaphragm, and the upper surface of the diaphragm becoming adherent to the structures in contact with it the abscess may open into the pleural cavity, or directly into the lung, or even into the pericardium. It is extremely difficult to distinguish between hepatic and subdiaphragmatic abscess, but if an opening has occurred into the lung, a careful examination of the pus that is being expectorated may be of great assistance in making the distinction. In the case of hepatic abscess it may be brick-red or reddish brown like anchovy sauce, and a microscopical examination may determine the presence of the *amœba coli*, although it should be noted that the latter is less often found in the pus than it is in the granulations scraped from the abscess wall. In the case of a suppurating hydatid cyst opening into the lung daughter cysts, shreds of laminated membrane, scolices or hooklets, may be found.

If the abscess arises from the outer surface or the upper part of the anterior surface marked bulging of the thoracic wall with obliteration or bulging of the intercostal spaces and widening of the epigastric angle on the affected side may be found. There may also be redness and œdema of the skin if the abscess is near the surface and about to discharge. In both conditions the liver may be enlarged and the edge felt a good way below the costal margin, the surface being smooth and usually tender.

If the abscess projects from the under surface or occurs in the region of the gall-bladder it may cause a local tender fluctuating swelling which may be mistaken for a distended gall-bladder.

If the abscess is situated in the left lobe it may give rise to a rounded prominence in the epigastric region and a smooth, tender, elastic, and possibly fluctuating tumour may be felt in connection with the liver there. Early in the disease, before adhesions have taken place, friction-sounds may be heard on auscultating over the affected area.

An abscess may open into various parts besides those already mentioned—into the stomach, the general peritoneal cavity, the duodenum or the colon.

The chief symptoms of an abscess of the liver are: a feeling of chilliness in spite of warm surroundings, rigors, intermittent pyrexia, profuse sweating attacks, rapid pulse, dull heavy pain in the right hypochondrium and right shoulder, vomiting, dry furred tongue, severe prostration and emaciation, a peculiar earthy sallowness of the complexion, and possibly jaundice. In some cases, however, the symptoms may be very obscure, or even entirely latent until some complication occurs, such as the opening of the abscess into the lung or one of the other places mentioned above. The rigors, sweating, and intermittent pyrexia may lead to a diagnosis of malaria, for nearly all the patients have been in malarial districts. A blood examination should help to differentiate these two diseases, for in the case of hepatic abscess there is nearly always a polymorphonuclear leucocytosis, whereas in malaria there would be at most only a slight temporary leucocytosis for a few hours after the initial rigor, and after that a diminution in the total number of leucocytes with a definite lymphocytosis; moreover, protozoa would probably be found in some of the red corpuscles. The administration of quinine may also be of service in making the differential diagnosis. For practical purposes an intermittent fever which resists quinine is not malaria.

